# Enhancing Clinical Decision-Making in Pediatric Monitoring: Learning Threshold Alarm Patterns to Predict Critical Illness

**DOI:** 10.3390/bioengineering12111210

**Published:** 2025-11-05

**Authors:** Christina Chiziwa, Mphatso Kamndaya, Patrick Phepa, IMPALA Project Team, Alick O. Vweza, Job Calis, Bart Bierling

**Affiliations:** 1 Department of Mathematical Sciences, School of Science and Technology, Malawi University of Business and Applied Sciences, Blantyre 309070, Malawi; mkamndaya@mubas.ac.mw (M.K.); pphepa@mubas.ac.mw (P.P.); 2 Department of Electrical Engineering, School of Engineering, Malawi University of Business and Applied Sciences, Blantyre 309070, Malawi; 3 IMPALA Project Study Team, IMPALA Project, Amsterdam Institute for Global Health and Development, 1105 BP Amsterdam, The Netherlands; 4 Department of Paediatric Intensive Care, Emma Children’s Hospital, Amsterdam UMC, 1105 AZ Amsterdam, The Netherlands; 5 Amsterdam Institute for Global Health and Development, 1105 BP Amsterdam, The Netherlands; 6 Department of Pediatrics and Child Health, Kamuzu University of Health Sciences, Blantyre BP 360, Malawi; 7 GOAL 3, 5223 AL Den Bosch, The Netherlands

**Keywords:** threshold alarm patterns, critical illness events, pediatric patients, machine learning

## Abstract

Background: Patient monitors assist caregivers in identifying deterioration earlier by using threshold alarms. Not all of the threshold alarms necessitate immediate action, but some are a result of the triggering of a physiological event. We aim to use pattern recognition techniques to identify threshold alarm signal patterns before the onset of critical illness, thereby enabling the faster and more effective detection of clinical deterioration and supporting better clinical decision-making. Method: Secondary data from 774 pediatric patients were extracted from the IMPALA Project conducted in the High Dependency Unit (HDU) at Queen Elizabeth and Zomba Central Hospitals in Malawi. The threshold alarm data were generated from the vital signs using WHO age cut-offs and GOAL3 age cut-offs. Time-segmented alarm analysis was conducted to examine the distribution of threshold alarms around each vital sign 8 h before the onset of critical illness events. Density-Based Spatial Clustering of Applications with Noise (DBSCAN) was used to generate threshold alarm signal patterns for each signal per individual before the onset of a critical illness event. We used three machine learning approaches, random forest, support vector machine, and decision tree, to learn threshold alarm patterns in signals preceding critical illness events. Results: The total threshold alarm summed up to (3,910,083) in total for WHO and (2,041,740) for GOAL3. Temporal distributions of ECGRR, ECGHR and oxygen saturation rate (SPO2) threshold alarms were observed, revealing patterns before the onset of the critical illness events. A pattern of most threshold alarms was distributed around (40–60) for ECGRR upper threshold alarms and (0–20) for ECGRR lower threshold alarms, (80–85) for ECGHR lower threshold alarms and (140–160) for ECGHR upper threshold alarms, and (85–90) for SPO2 for death (CPR and PICU), around WHO threshold alarms. For sepsis, most of these threshold alarms were distributed around (40–50) of ECGRR upper threshold alarms and (0–20) for ECGRR lower threshold alarms, (150–180) for ECGHR upper threshold alarms, and (85) for SPO2 for WHO threshold alarms, and most of the threshold alarms had a duration of less than 30 s. The results indicate that the random forest classifier performed better in learning the threshold patterns, with an accuracy of 93% and an area under the curve of 92, compared to using the support vector machine learning model and decision tree, which had an accuracy from a classification report of 85% and 94%, with low death and sepsis precision, recall, and F1-Score. Conclusions: The analysis of threshold alarm data before critical illness events has provided valuable insights into threshold alarm patterns associated with death and sepsis. The data revealed distinct patterns in ECGRR, ECGHR, and SPO2 signals, and most of the threshold alarms were in the lower duration. The random forest classifier effectively distinguished these learned patterns around death and sepsis events compared to other algorithms. Further studies are required on the use of algorithms on all vital sign signal features in clinical settings.

## 1. Introduction

Children can deteriorate rapidly. To ensure early deterioration identification and early intervention to prevent mortality and morbidity, patient monitors have been designed for continuous vital sign monitoring in intensive care units (ICUs) and emergency rooms [1,2,3,4,5,6]. They continuously monitor the patient’s vital signs, detect any changes, and notify the caregivers for immediate intervention through a threshold alarm [7]. In most traditional patient monitors, the continuous patient monitoring systems start their alarms when a vital sign goes above or below a predetermined threshold. These threshold alarms help caregivers to either attend to emergencies or to flag deterioration [8,9]. Not all threshold alarms require action because some threshold alarms that signal deterioration are often false, not indicating deterioration but rather transient physiological events such as crying, drinking, and normal coughing [10,11,12]. These non-actionable threshold alarms may overwhelm to caregivers, affecting the effectiveness of the use of the patient monitors, and can lead to the death of children [13,14,15]. Despite various efforts to mitigate non-actionable threshold alarms—such as implementing alarm delays, adjusting alarm thresholds, and personalizing settings for individual patients to focus on threshold alarms that signify deterioration—we still have numerous non-actionable alarms that overwhelm caregivers and affect the effectiveness of the patient monitors in detecting deterioration [16,17,18,19,20,21,22,23,24,25]. Deterioration may be detected more intelligently, more accurately, and possibly earlier by detecting patterns in these threshold alarms in vital sign signals [26,27]. Artificial Intelligence methods assist caregivers in recognizing these patterns, simplifying monitoring and reducing workload [27,28]. These methods have been poorly studied in general and have not been developed at all for lower-resource settings [29]. Therefore, we aim to utilize pattern recognition techniques to learn these threshold alarm signal patterns before the onset of critical illness events and provide an innovative approach to help enhance clinical decision-making in pediatric monitoring by encouraging caregivers to pay more attention to monitor alarms.

This study introduces a novel, data-driven approach to optimizing pediatric patient monitoring by employing machine learning techniques to identify threshold alarm patterns preceding critical illness events. Traditional patient monitoring systems depend on fixed threshold alarms, often resulting in excessive non-actionable alerts that contribute to the ineffectiveness of patient monitoring. Therefore, this research innovatively used Density-Based Spatial Clustering of Applications with Noise (DBSCAN) to detect threshold alarm patterns before the onset of critical illness events and three machine learning approaches—random forest, support vector machine, and decision tree—to learn threshold alarm patterns in signals to predict critical illness events. This helped enhance clinical decision-making in pediatric monitoring by encouraging caregivers to pay closer attention to monitoring alarms.

## 2. Methods

### 2.1. Data Source—The IMPALA Project

**Figure 1:**
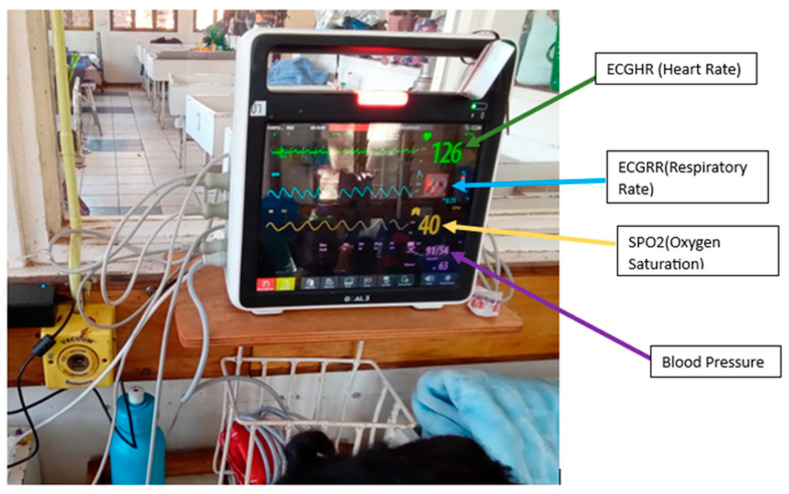
IMPALA patient monitor device from which we extracted our vital signs data.

Data from pediatric patients aged 1 month to 5 years with continuous vital sign recordings for at least 24 h were selected for analysis. The cohort consisted of 774 patients and was stratified into three age groups: neonates, infants, and pediatric children. This secondary data was extracted from the IMPALA patient monitoring device, which is a state-of-the-art monitoring solution that was utilized in the High Dependency Unit (HDU) at Queen Elizabeth and Zomba Central Hospitals in Malawi under the IMPALA Project to enhance an ecosystem of early deterioration detection in clinical settings. The IMPALA project is a consortium of the Amsterdam Institute for Global Health & Development (Amsterdam, The Netherlands), GOAL3 (Den Bosh, The Netherlands), Malawi University of Business and Applied Sciences (Blantyre, Malawi), Kamuzu College of Health Sciences (Blantyre Malawi), Imperial College London (South Kensington, London, UK), The National eHealth Living Lab. (Leiden, The Netherlands), and Training Unit of Excellence (Zomba, Malawi). The IMPALA Project is working to address the problem of mortality rate by detecting vital sign deterioration at an early stage in patients’ vital signs using the IMPALA patient monitors. This IMPALA patient monitor device, as shown in Figure 1, is specifically designed to capture and store real-time vital sign data, offering a comprehensive overview of a patient’s physiological parameters. These vital sign parameters include ECG heart rate, blood pressure, ECG respiratory rate, oxygen saturation (SPO2), SPO2HR, and temperature, which are all essential for monitoring patients in critical conditions. The parameters were recorded every second, creating a rich time series dataset. For our study, we focused on analyzing the following vital signs for threshold alarm analysis: ECG heart rate (ECGHR), ECG respiratory rate (ECGRR), and oxygen saturation (SPO2). We also used critical illness event data, which we extracted from the clinical group and annotated using RedCap, and the data included patient ID, critical illness event (CIE), and the date and time of its occurrence.

Ethical clearance and permission for conducting this study were under the main IMPALA Project study protocol approved by the College of Medicine Research Ethics Committee (COMREC REF. Number: P.01/22/3552). Patient confidentiality and privacy were maintained during data extraction and analysis.

### 2.2. Threshold Alarm Generation and Summary Statistics

**Figure 2:**
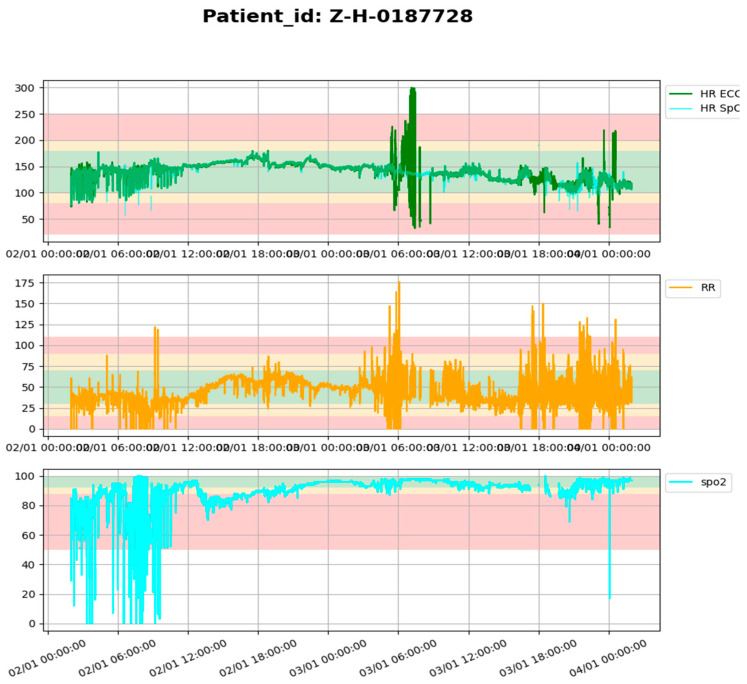
Patient vital sign data recorded during the monitoring period. *HR* represents ECGHR, *RR* represents ECGRR, and *spo*2 represents SPO2.

**Figure 3:**
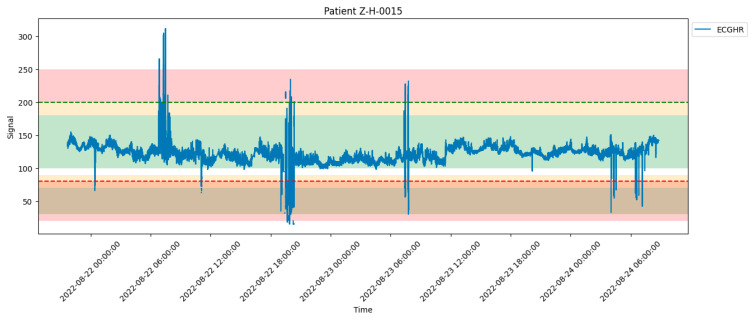
Visualization of ECGHR signal distribution for the patient throughout their 24-h monitoring period; we generated our threshold alarms using the pre-determined age cut-off threshold. The dash lines represent the threshold cut offs.

From 774 participants, we had 100,000,000 rows of vital signs for all the patients within their monitoring period, which was more than 24 h of continuous monitoring. The vital sign signal data of some signals are presented in Figure 2 below. We used Python (Version: 3.12.2, Python environment: Jupiter, VS Code (Raymond, WA, USA)), pandas dataframe functions to drop and replace non-physiological values with nan in the time series vital sign measurement data of the patients. The functions were used in aspects where there is no data due to sensor disconnection in the time series data of the vital sign measurements of the patients. The cleaned patient time series vital sign data were used to retrospectively generate threshold alarms using World Health Organization age cut-offs and GOAL3 age cut-offs, which are shown in Table 1. We divided the patients into age groups and used each age group to generate threshold alarms using age cut-offs from WHO and GOAL3, as shown in Table 1. And the threshold alarms were generated such that, if any vital signs crossed the predetermined upper and lower age cut-off thresholds of the vital signs, it was considered a threshold alarm, as shown in Figure 3. The starting and ending time, which means the time the signal (SPO2, ECGHR, or ECGRR) went above the threshold or below the threshold, duration (which is the difference between the starting point and the end time in seconds), signal of the vital signs, and value of that threshold were appended to the threshold alarm data frame. The age cut-off algorithm used is shown below: https://github.com/Christina-Chiziwa/AGE-THRESHOLD_ALARM/tree/main (accessed on 6 July 2020, made available upon request).

### 2.3. Time-Segmented Threshold Alarm Analysis

We used time series analysis to identify the distribution of alarms within an 8 h window. A scatter plot was used to visualize the temporal distribution of threshold alarms before the occurrence time of the critical illness events. We looked at the time the critical illness events occurred, 8 h before the critical illness events occurred, and what was happening to the distribution of the threshold alarms. And this 8 h period was divided into 2 h to create four time segments from the 8 h window.

### 2.4. Density-Based Spatial Clustering of Applications with Noise (DBSCAN)

We used Density-Based Spatial Clustering of Applications with Noise (DBSCAN) to identify and cluster threshold alarm patterns. The DBSCAN clustered in the dataset the most frequent and common threshold values of SPO2, ECGHR, and ECGRR for patients who died (which included CPR and PICU) and had sepsis by looking at 8 h before the occurrence time of critical illness events. We also observed the common threshold values of SPO2, ECGHR, and ECGRR for patients without critical illness events. We chose to utilize the DBSCAN algorithm due to its robustness in handling noisy and irregular data. The Euclidean distance of core points, which is the number of thresholds in that clustering, was 10, and DBSCAN signal processing is shown in the code below: https://github.com/Christina-Chiziwa/DBSCAN-and-Machine-learning-models/tree/main (accessed on 6 July 2020, made available upon request). Our Equation (1) mathematically represents the code.

(1) $$Dist\left(P,Q\right)=\sqrt{\sum_{i=1}^{d}{\left(P_{i}-Q_{i}\right)}^{2}}$$

*d* is the dimensionality of the data. $P_{i}\;and\;Q_{i}$ are the *i*-th coordinates of *P* and *Q*. The core point condition is given by the following equation:

$$CorePoint:\left|N_{\varepsilon }(P)\right|\geq minPts$$

where $\left|N_{\varepsilon }(P)\right|$ denotes number of points of the ε−neighborhood.

### 2.5. Machine Learning Models

After identifying, clustering, and generating patterns from DBSCAN data for patients with death or sepsis and those without critical illness events (CIEs), the key extracted features included patient ID, physiological signal type, CIE status (either sepsis, death/CPR/PICU), the most frequent alarm threshold in each group, and the duration of these alarm patterns. These features captured the structure of threshold alarm patterns used by the models (Table 2), as illustrated in Equation (2) and the code below: https://github.com/Christina-Chiziwa/DBSCAN-and-Machine-learning-models/tree/main (accessed on 11 July 2023, made available upon request). We trained three machine learning models—random forest classifier (n_estimators = 200, criterion = “gini”, max_depth = None, random_state = 42), a support vector machine (SVM) (kernel = “rbf”, C = 1.0, gamma = “scale”, probability = True, random_state = 42), and a decision tree (criterion = “gini”, max_depth = None, random_state = 42)—to learn the generated patterns and compare performance across interpretability, robustness, and classification power, thereby providing a balanced evaluation of their suitability in pediatric monitoring.

The threshold alarm pattern dataset was balanced, consisting of 119 patients with sepsis and death (combined with PICU and CPR) and 119 patients without any of these CIEs. The dataset was then split into training and testing sets using a 70/30 train–test split strategy. To account for repeated measures, we ensured that no patient appeared in both sets. The model was then tested on the remaining patients who were not part of the training set. Model performance was evaluated using a classification report, providing precision, recall, F1-score, and the area under the curve (AUC).

(2) $$P\left(y|x\right)=\frac{1}{N}\sum_{i=1}^{N}T_{x}\;\left(x\right)=Mode(\;T_{1}\left(x\right),\;T_{2}\left(x\right),\ldots ,T_{N}\left(x\right))$$

## 3. Results

### 3.1. Data Summary

A total of 774 pediatric patient participants were used, with 100,000,000 rows of vital sign data from which the threshold alarms were generated using different age cut-offs (Table 1). The threshold patterns were generated from all the participants, including 119 patients, consisting of 46 sepsis participants and 73 death participants (which included CPR and PICU) (Table 3). This helped us to have a rich dataset on patient characteristics, which included control variables.

#### Summary Statistics of Threshold Alarms Generated Using WHO and GOAL3 Threshold Age Cut-Offs for All the Patients

From 100,000,000 vital sign data points, using the age–alarm threshold algorithm shared, we generated 3,910,083 threshold alarms using the WHO age cut-off, while the GOAL3 threshold age cut-offs produced 2,041,740 alarms (Table 4). The analysis shows that, by using WHO threshold age cut-offs, you have alarms that capture everything, including non-actionable alarms, while with the GOAL3 threshold age cut-off alarms capture more abnormalities. This comparison also helped identify which age-based thresholds were more effective and efficient with our learning algorithm to predict critical illness events, and the algorithm performed better using the WHO age cut-offs than the GOAL3 age cut-offs, providing sufficient data points for more the robust learning and prediction of critical illness events.

### 3.2. Time-Segmented Alarm Analysis

**Figure 4:**
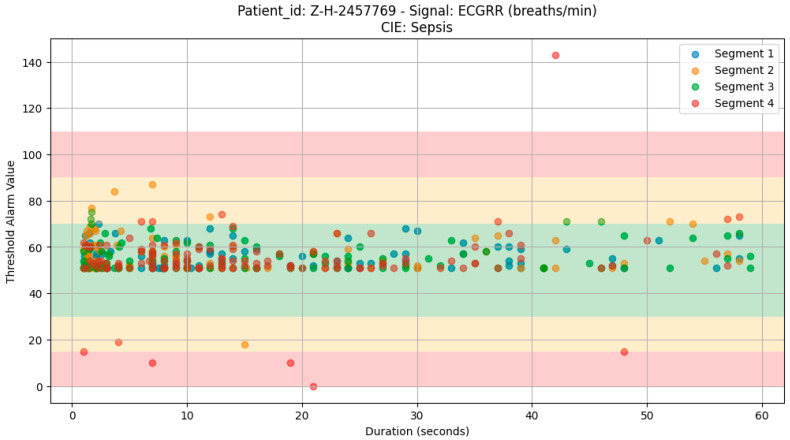
Visualization of ECGRR threshold alarms for patient Z-H-2457769 in four segments representing 2 h from the 8 h window before the occurrence of a critical illness event.

The analysis using a scatter plot showed that there was a distribution of noticeable threshold alarms within each segment of the 8 h window, from which we can detect and generate the threshold patterns to be used in our learning algorithm. We also discovered that the durations of most of these threshold alarms were below 30 s (Figure 4).

### 3.3. Density-Based Spatial Clustering of Applications with Noise (DBSCAN)

After identifying, clustering, and generating threshold alarm patterns for critical illness events using both the dataset using the WHO age threshold cut-offs and the GOAL3 age threshold cut-offs, we determined the following. For death (CPR and PICU), most WHO threshold alarm patterns were distributed as follows: ECGRR, upper 40–60 and lower 0–20; ECGHR, lower 80–85 and upper 140–160; and SPO2, 85–90. For GOAL3, they clustered as follows: ECGRR, upper 50–70 and lower 0–20; ECGHR, lower 70–85 and upper 150–175; and SPO2, 90.

**Figure 5:**
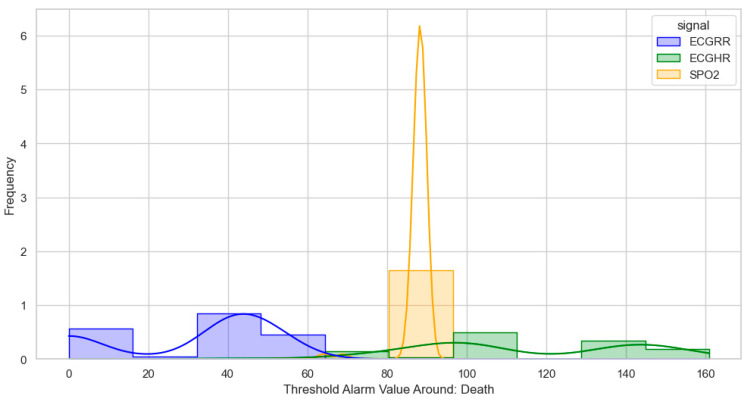
Distribution of threshold alarms for death/CPR/PICU from WHO threshold alarms.

**Figure 6:**
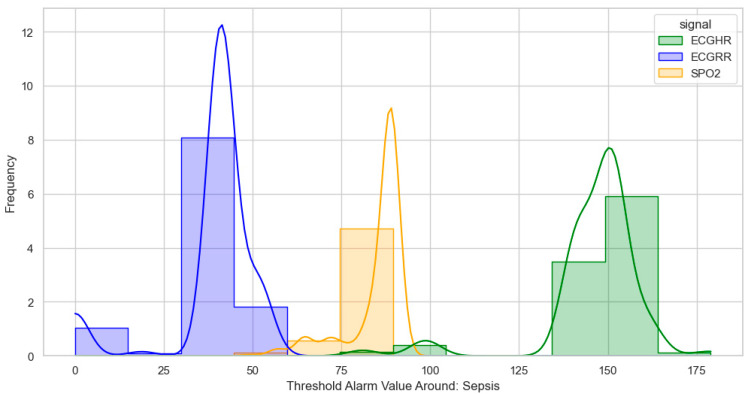
Distribution of threshold alarms for sepsis from WHO threshold alarms.

**Figure 7:**
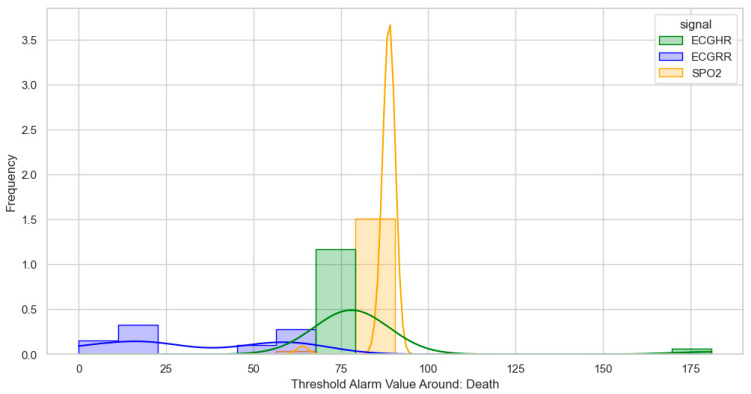
Distribution of threshold alarms for death/CPR/PICU from GOAL3 threshold alarms.

**Figure 8:**
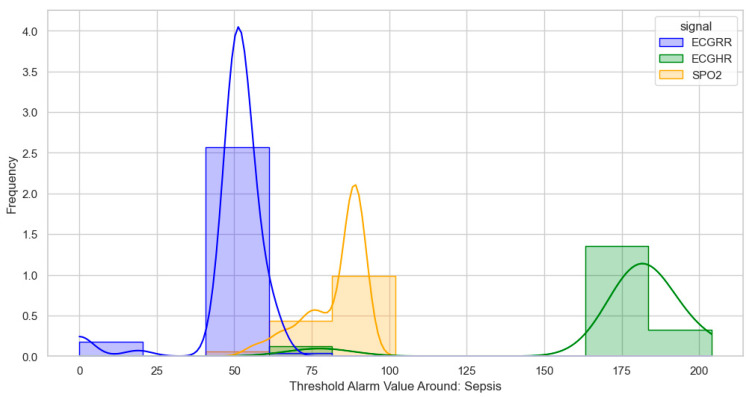
Distribution of threshold alarms for sepsis from GOAL3 threshold alarms.

For sepsis, the WHO alarms were mainly as follows: ECGRR, upper 40–50 and lower 0–20; ECGHR, upper 150–180; and SPO2, 85. GOAL3 alarms were as follows: ECGRR, upper 40–50 and lower 0–20; ECGHR, upper 170–200; and SPO2, 90. As shown in Figure 5, Figure 6, Figure 7 and Figure 8, most of these alarms occurred within durations under 30 s.

### 3.4. Model Performances

**Figure 9:**
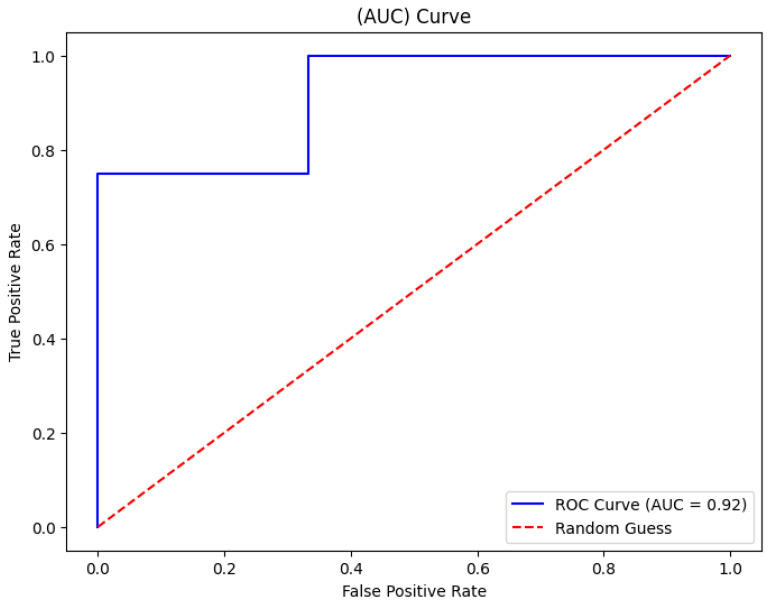
Area under the curve for random forest classifier evaluation.

**Figure 10:**
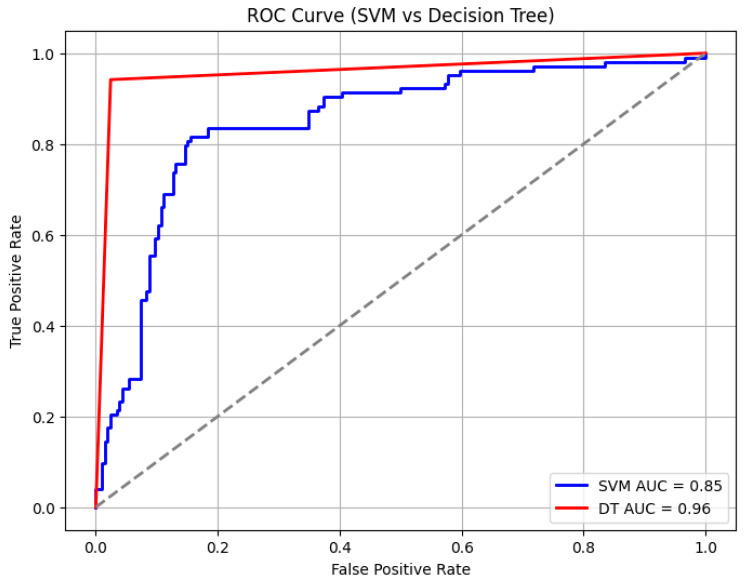
Area under the curve for support vector machine learning evaluation under WHO thresholds.

The random forest algorithm showed a strong performance in learning threshold alarm signal patterns. For WHO thresholds, it achieved a 93% accuracy with an AUC of 92% (Figure 9). For GOAL3 thresholds, its accuracy was 90%. Under WHO thresholds, death prediction (including CPR and PICU) had a precision of 0.90, a recall of 0.88, and an F1-score of 0.89, while sepsis prediction reached a precision of 0.91, a recall of 0.92, and an F1-score of 0.91. For GOAL3, death prediction recorded a precision of 0.90, a recall of 0.80, and an F1-score of 0.89, and sepsis prediction achieved a precision of 0.88, a recall of 0.91, and an F1-score of 0.93 (Table 5). In comparison, the support vector machine (SVM) and decision tree (DT) algorithms achieved AUCs of 85% and 96%, with overall accuracies of 85% and 94%, respectively (Figure 10 and Table 6). However, the SVM and DT showed a reduced precision and recall for death and sepsis due to the difficulty of handling noisy and irregular alarm data for the SVM, while the DT’s high accuracy was mainly due to overfitting, limiting its ability to generalize to new patterns. Among the three algorithms, random forest consistently outperformed SVM and decision tree in balancing accuracy, AUC, and class-specific performance.

## 4. Discussion

This study set out to enhance clinical decision-making in pediatric monitoring by learning threshold alarm patterns that could predict critical illness events (CIEs), specifically death/CPR/PICU and sepsis, using data from patient monitors. We evaluated WHO and GOAL3 age-based cut-offs, integrating temporal and spatial analyses with machine learning to identify alarm configurations that could support an earlier recognition and intervention in deteriorating patients.

Our findings show that the WHO threshold cut-offs generated a higher overall volume of alarms compared to the GOAL3 cut-offs. It should be noted that alarm limits serve a different purpose, as they are primarily used to signal an acute need to review the patient and provide emergency treatments. While the WHO settings may capture more events, the GOAL3 cut-offs tended to identify alarms that were more clinically significant, suggesting a potential role in reducing alarm fatigue and directing caregiver attention to high-priority cases. This study highlights that the more “subtle” deviations of vital signs, as detected by the WHO thresholds, hold important information in detecting overall deterioration, and thus should be used in algorithms to detect this.

Temporal analysis revealed that alarms clustered most heavily within the two hours preceding CIEs, with distinct signal patterns for ECGRR, SPO2, and ECGHR. Most alarms were short in duration, rarely exceeding 30 s, which may influence how caregivers interpret their urgency. Spatial clustering using DBSCAN identified clear signal-specific value ranges predictive of death or sepsis: for example, ECGRR upper thresholds of 40–60 and ECGHR values of 140–160 for death under WHO settings, and ECGHR upper thresholds of 170–200 for sepsis under GOAL3.

Among the tested algorithms, the random forest classifier demonstrated the strongest predictive performance, achieving a 92% accuracy (AUC 0.92) with WHO thresholds and slightly lower but still robust results with GOAL3 thresholds. The model achieved a particularly high precision and recall for sepsis predictions. This is because it is an ensemble method that aggregates the outputs of multiple decision trees, reducing overfitting and improving generalizability. This robustness was particularly important given the noisy and irregular nature of ICU threshold alarm data. The random forest classifier effectively captures complex, non-linear interactions between alarm features, and, finally, the random forest classifier maintains a relatively high interpretability by enabling feature importance analysis, which helps identify which alarms contributed most to the prediction. By combining interpretability, robustness, and strong classification power, the random forest model provided a balanced and reliable evaluation of threshold alarm patterns in pediatric monitoring, explaining its superior performance compared to support vector machines and decision trees, which performed less consistently, producing lower accuracy and less balanced precision–recall metrics.

These results are consistent with earlier work by Hu et al. [22] and others [17,23], which showed that identifying combinations of alarm patterns can improve the early recognition of deterioration beyond single-threshold monitoring. Importantly, our study extends this approach to pediatric care in lower-resource settings, where AI-driven monitoring tools remain largely unexplored.

A key strength of this study lies in its integration of time series, clustering, and supervised learning approaches to analyze both temporal and spatial alarm patterns. By directly comparing WHO and GOAL3 thresholds, we provide context-specific insights into how alarm configurations influence the balance between sensitivity and specificity. The dual focus on temporal clustering before CIEs and signal-specific spatial patterns offers a more nuanced understanding of alarm dynamics, increasing the potential for targeted, real-time interventions.

The analysis was restricted to three vital signs (ECGRR, SPO2, ECGHR), excluding potentially informative parameters such as blood pressure or temperature. Additionally, model validation was performed internally, and external validation across multiple sites is required to confirm generalizability. Finally, while predictive patterns were successfully identified. Future research should focus on real-world integration into patient monitors and include all the vital signs.

## 5. Conclusions

The analysis provided valuable insights into alarm patterns linked to critical illness events, specifically death and sepsis. By leveraging a DBSCAN clustering algorithm, we identified meaningful spatial patterns in the distribution of alarms based on the signal threshold value and the duration during which they are mostly distributed. For example, distinct threshold ranges were observed for ECGRR, ECGHR, and SPO2 signals preceding death and sepsis, revealing actionable patterns that could guide clinical responses. The random forest classifier proved highly effective in identifying these patterns, especially under WHO threshold alarms, achieving an accuracy of 93% for death and sepsis predictions, with consistently high precision, recall, and F1 scores. Of course, the algorithm performed better using GOAL3 threshold alarms, with a 90% accuracy and with minimal and manageable alarms, which can help in directing caregivers’ attention to the threshold alarms, since there will be a manageable number of alarms. The random forest classifier demonstrated a greater robustness and practicality for real-time monitoring applications, particularly in settings with limited technology resources, compared to other models. Implementing such models on patient monitors could empower caregivers to make timely interventions and decisions, ultimately improving patient outcomes by prioritizing early detection and response to critical illness events. However, further research is needed to refine it into a prediction model that includes all vital signs.

## Figures and Tables

**Table 1:** Age cut-offs for different age groups from WHO and GOAL 3, which were used to generate threshold alarms in each group.

		ECGHR UPPER (Beats per Minute (bpm))	ECGHR LOWER (Beats per Minute (bpm))	SPO2 (Oxygen Saturation%)	ECGRR UPPER (Breaths/min)	ECGRR LOWER (Breaths/min)
WORLD HEALTH ORGANIZATION (WHO) Cut-offs						
Age Group (Neonatal, infant, and pediatric)	<1 year	160	100	90	50	20
	1–3 years	150	90	90	40	10
	3–4 years	140	80	90	40	10
GOAL3 Cut-offs						
Age Group (Neonatal, infant, and pediatric)	<1 year	200	80	90	60	20
	1–3 years	180	80	90	50	20
	3–4 years	170	70	90	50	20
	>4 years	170	70	90	40	20

**Table 2:** The algorithm features used in the study and their descriptions.

Algorithm, Threshold Alarm Pattern, Features Used	Description of the Feature
Signals	ECGRR, ECGHR, and SPO2
Actual value of an alarm	ECGRR upper, ECGRR lower, SPO2, ECGHR upper, and ECGHR lower alarm value
Duration of an alarm	In seconds
Critical illness events (CIE)	Death (which included PICU and CPR), sepsis
Similar threshold alarm values	Patients without critical illness events Patients with critical illness events
Time to (CIE) occurrence	8 h window

**Table 3:** Patient data characteristics and their percentages (N = 774).

Patient Data Characteristics		Frequency	Percentage
Age	Neonatal (<1 month)	1	0.14%
	Infants (1 month–11 months)	330	42.61%
	Pediatric (1 year–5 years)	442	57.25%
Gender	Male	445	58%
Groups of patients	Patients with CIE	240	31%
	Patient without CIE	533	69%
CIE (Death, CPR, PICU, and Sepsis n = 119)	Death and CPR	54	45%
	PICU	19	16%
	Sepsis	46	39%
CIE (others n = 274)	Blood transfusion	57	21%
	Bronchodilator support	14	5%
	Coma	16	6%
	Convulsion	55	20%
	Respiratory support	92	34%
	Malaria treatment	5	1%
	Intravenous fluid bolus	18	7%
	ICU	12	4%
	IV or enteral	5	1%
	Inotropic support	3	1%

**Table 4:** Summary threshold alarms using different age cut-offs for all the patients (N = 774).

Organization Age Cut-Offs		Total Threshold Alarms	Total Percentage of Each Patient Group from Total Threshold Alarms	ECGHR Alarms from the Total Alarms	ECGRR Alarms from the Total Alarms	SPO2 Alarms from the Total Alarms
WHO age cut-off		3,910,083		1,385,758	2,258,952	265,373
	WHO Patient (N = 533, Without CIE) Threshold Alarms	2,189,647	56%	459,826 (21% of total patients without CIE alarms)	1,554,649 (71% of total patients without CIE alarms)	175,172 (8% of total patients without CIE alarms)
	WHO Patient (N = 240, With CIE) Threshold Alarms	1,720,436	44%	361,292	1,221,510	137,634
GOAL3 age cut-off		2,041,740		409,617	1,429,633	202,490
	GOAL3 Patient (N = 533, Without CIE) Threshold Alarms	1,143,374	56%	248,109	811,796	83,468
	GOAL3 Patient (N = 240, With CIE) Threshold Alarms	898,366	44%	188,657	637,840	71,869

**Table 5:** Classification report for the algorithm in predicting death and sepsis.

Organization Threshold Alarms	CIE	Precision	Recall	F1-Score
World Health Organization threshold alarms	Death, CPR, and PICU	0.90	0.88	0.89
	Sepsis	0.91	0.92	0.91
Accuracy 0.92				
GOAL3 threshold alarms	Death, CPR, and PICU	0.90	0.81	0.85
	Sepsis	0.88	0.91	0.89
Accuracy 0.90				

**Table 6:** Classification report for the decision tree and SVM algorithm in predicting death and sepsis.

Models	CIE	Precision	Recall	F1-Score
Decision tree	Death, CPR, and PICU	0.89	0.88	0.89
	Sepsis	0.90	0.89	0.90
Accuracy 0.94				
Support vector machine learning algorithm	Death, CPR, and PICU	0.85	0.59	0.72
	Sepsis	0.72	0.97	0.83
Accuracy 0.85				

## Data Availability

The data presented in this study are available on reasonable request from the corresponding author and the IMPALA Project Team. The data are not publicly available due to privacy and ethical restrictions involving human participants.

## Abbreviations

The following abbreviations are used in this manuscript:

ECHHR	Electrocardiography heart rate
ECHRR	Electrocardiography respiratory rate
SPO2	Oxygen saturations
